# RecA deletion disrupts protein homeostasis, leading to deamidation, oxidation, and impaired glycolysis in *Cronobacter sakazakii*

**DOI:** 10.1128/aem.01971-24

**Published:** 2024-12-31

**Authors:** Ping Lu, Juan Xue, Xuemeng Ji

**Affiliations:** 1 Tianjin Key Laboratory of Ophthalmology and Visual Science, Tianjin Eye Institute, Tianjin Eye Hospital 159421 , Tianjin, Tianjin, China; 2 Nankai University Affiliated Eye Hospital, Nankai University, Tianjin, China; 3 Institute of Infection and Immunity, Taihe Hospital, Hubei University of Medicine 74765 , Shiyan, Hubei, China; 4 School of Medicine, Nankai University 481107 , Tianjin, Tianjin, China; The Pennsylvania State University, University Park, Pennsylvania, USA

**Keywords:** *Cronobacter sakazakii*, RecA, virulence factors, desiccation resistance, biofilm formation, protein deamidation, protein oxidation, glycolysis impairment

## Abstract

*Cronobacter sakazakii* is a foodborne pathogen linked to severe infections in infants and often associated with contaminated powdered infant formula. The RecA protein, a key player in DNA repair and recombination, also influences bacterial resilience and virulence. This study investigated the impact of *recA* deletion on the pathogenicity and environmental stress tolerance of *C. sakazakii* BAA-894. A *recA* knockout mutant displayed impaired growth, desiccation tolerance, and biofilm formation. In a rat model, the mutant demonstrated significantly reduced virulence evidenced by higher host survival rates and lower bacterial loads in blood and tissues compared to the wild-type strain. Proteomic analysis revealed extensive disruptions in protein expression, particularly downregulation of carbohydrate metabolism and respiration-related proteins, alongside increased protein deamidation and oxidation. Functional assays identified fructose-bisphosphate aldolase as a target of oxidative and deamidative damage, resulting in reduced enzymatic activity and glycolytic disruption. These findings highlight the critical role of RecA in maintaining protein homeostasis, environmental resilience, and pathogenicity in *C. sakazakii*, providing valuable insights for developing targeted interventions against this pathogen.

**IMPORTANCE:**

*Cronobacter sakazakii* poses significant risks due to its ability to persist in low-moisture environments and cause severe neonatal infections. This study identifies RecA as a key factor in environmental resilience and virulence, making it a promising target for mitigating infections and contamination. Inhibiting RecA function could sensitize *C. sakazakii* to stress during production and sterilization processes, reducing its persistence in powdered infant formula. Future research on RecA-specific inhibitors may lead to innovative strategies for enhancing food safety and preventing infections caused by this pathogen.

## INTRODUCTION

*Cronobacter sakazakii*, formerly known as *Enterobacter sakazakii* until its reclassification in 2007, is recognized as an opportunistic pathogen that primarily infects neonates under 6 months of age and elderly individuals (1, 2). Infections in neonates often result in life-threatening conditions, such as necrotizing enterocolitis, neonatal meningitis, and septicemia, with mortality rates ranging from 40 to 80% (3). *C. sakazakii* possesses several mechanisms to invade host tissues and establish infections. It induces intracellular bacterial endocytosis to enter human brain microvascular endothelial cells, promotes inflammation and cell apoptosis, and disrupts tight junctions to facilitate bacterial translocation, all of which contribute to its pathogenicity (4–6).

*C. sakazakii* exhibits exceptional environmental resilience, particularly in low-moisture food products like powdered infant formula. This bacterium surpasses the desiccation tolerance of other foodborne pathogens, such as *Salmonella*, *Listeria monocytogenes*, and *Escherichia coli* (7, 8). Remarkably, *C. sakazakii* can survive for over 2.5 years in powdered infant formula (8). Epidemiological studies have established a strong link between infections in infants and contamination of infant formula, leading to its classification as a Category A pathogen in powdered infant formula by the Food and Agriculture Organization and the World Health Organization (9).

The pathogenicity of *C. sakazakii* is associated with a wide range of virulence factors. Proteins, such as outer membrane protein A (OmpA) and OmpX, facilitate bacterial invasion and intracellular replication (10, 11). Other factors, including NlpD, DnaK, and Yrt2a, enhance acid tolerance and pathogenicity (12–14), while the *ptsH* gene plays a role in regulating carbon metabolism, stress response, and virulence (15). Additionally, proteins like ZpX, Cpa, and type III hemolysin Hly are crucial for host cell deformation, serum resistance, and hemolytic activity (16, 17). Regulatory proteins, such as Hfq, which influences outer membrane protein synthesis, also contribute significantly to virulence (14). The roles of various lipoproteins (*slyB*, *blc*, *tolC/A*), flagellar-related genes (*flhD*, *motA*, *flgM*, *flgB*, *fliC*), and regulatory systems like CpxAR and SdiA in *C. sakazakii*’s virulence have been extensively studied (6, 18–20).

RecA, a key enzyme in homologous recombination and DNA repair, is vital for maintaining genomic integrity under stress conditions. Its role in facilitating the strand exchange between single- and double-stranded DNA is well-documented (21). In *E. coli*, the plasmid expression of *Deinococcus radiodurans* RecA confers protection against UV-A exposure (22, 23). However, the role of RecA in desiccation tolerance remains unclear. Additionally, while RecA’s involvement in virulence has been confirmed in several bacterial species, such as *Acinetobacter baumannii*, *Pasteurella multocida*, *Riemerella anatipestifer*, and *Porphyromonas gingivalis* (24–27), its role in other species like *Xenorhabdus bovienii* appears to be negligible (28).

Despite its established importance in DNA repair and stress responses, the role of RecA in the environmental resilience and virulence of *C. sakazakii* remains poorly understood. This study aims to bridge this knowledge gap by examining the effects of RecA deletion on *C. sakazakii*, with a particular focus on its role in modulating protein deamidation, oxidative modifications, and glycolysis. A proteomic approach was employed to elucidate how RecA deficiency disrupts protein homeostasis, thereby impairing bacterial growth, environmental stress tolerance, and virulence. The findings provide new insights into the molecular underpinnings of *C. sakazakii*’s adaptability and offer potential targets for developing effective strategies to mitigate food contamination risks associated with this pathogen.

## RESULTS

### Construction and growth characteristics of the RecA mutant in *C. sakazakii*

**Fig 1:**
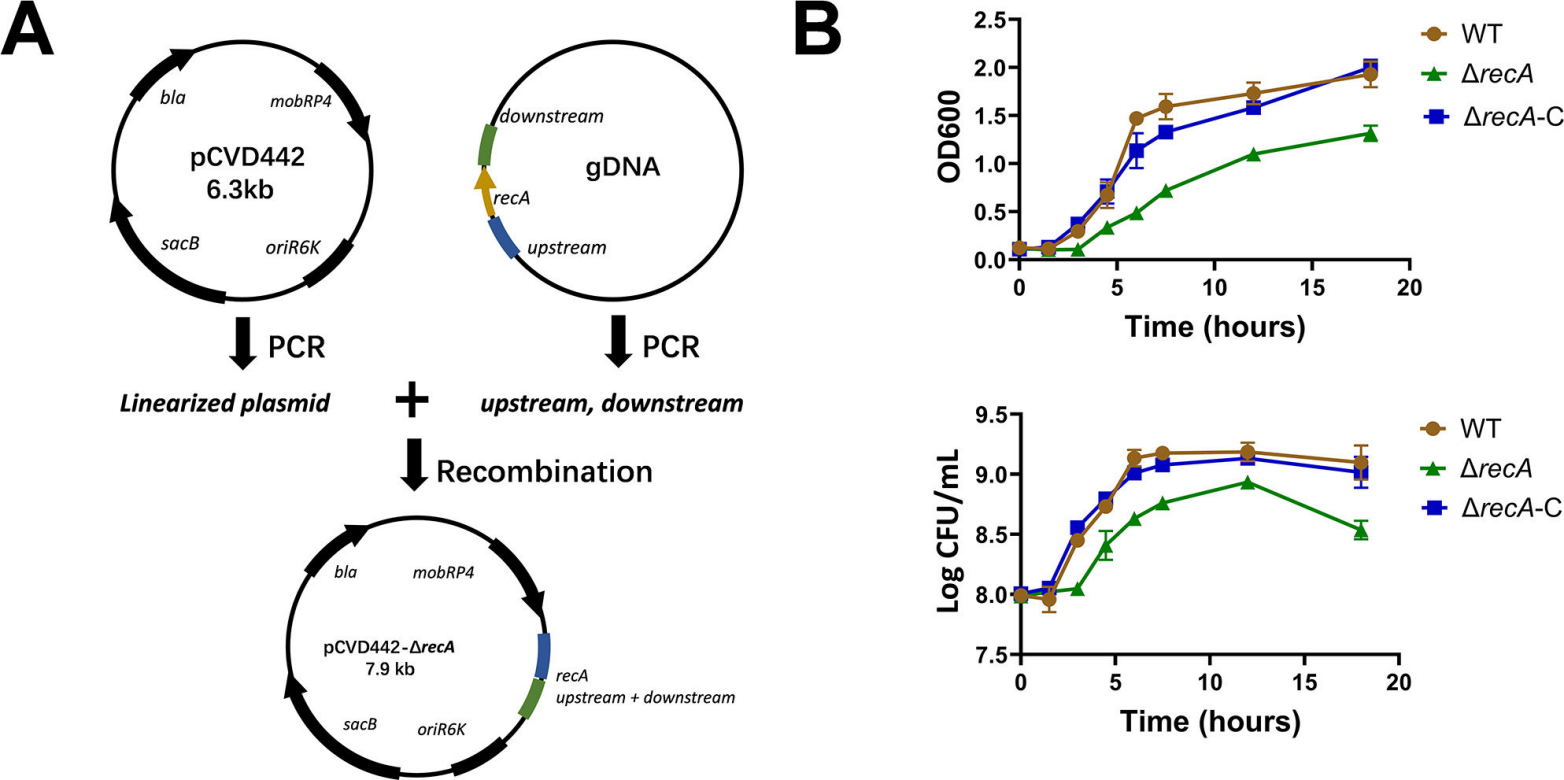
Construction and growth characteristics of the *reca* mutant in *C. sakazakii*. (**A**) Creation of the pCVD442 suicide plasmid for the deletion of *recA* in *C. sakazakii*. (**B**) Growth of the wild-type (WT), Δ*recA*, and complemented Δ*recA* strain (Δ*recA*-C) in LB medium at pH 7.0. Bacterial growth was monitored by measuring the optical density at 600 nm (OD600) over time, while viable cell count was determined by colony-forming units at various time points. The data points presented were the average and standard deviation from three biological replicates.

To evaluate the role of the *recA* gene in the environmental resilience and pathogenicity of *C. sakazakii* BAA-894, a *recA* knockout mutant was created using the pCVD442 suicide plasmid (Fig. 1A). Sequencing confirmed the successful deletion of the gene. When cultivated in LB medium at 37°C, the mutant displayed significantly reduced growth rate compared to the wild-type strain. This growth deficiency was effectively restored by reintroducing a *recA* complementation plasmid (Fig. 1B). These results indicate that the *recA* gene was crucial for maintaining the normal growth rate of *C. sakazakii*.

### RecA knockout reduces bacterial virulence

**Fig 2:**
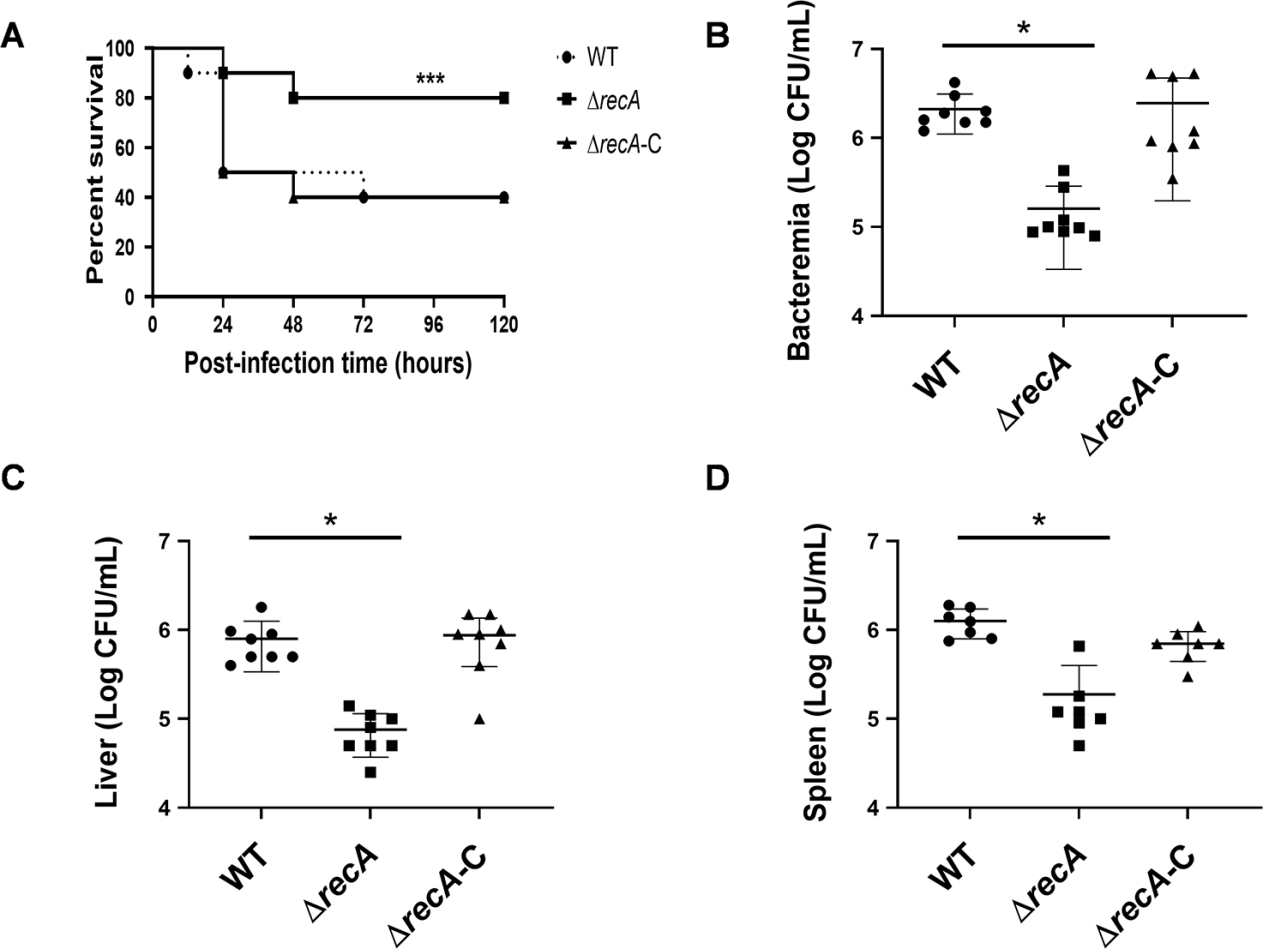
Impact of a *recA* knockout on bacterial virulence. (**A**) Survival curves of rat pups following oral infection with wild-type, *recA* knockout, and complemented strains, with data pooled from multiple experiments. Statistical significance was determined using the log-rank test (*P < 0.05; **P < 0.01; ***P < 0.001) for comparison between the survival curves of wild-type and *recA* knockout strains. (**B–D**) Quantification of colony-forming units (CFUs) in the blood, liver, and spleen of infected rats 24 h post-infection, as determined by organ homogenization and plating.

To investigate the role of *recA* genes in the pathogenicity of *C. sakazakii* BAA-894, rats were infected with the wild-type strain, the Δ*recA* mutant, and the Δ*recA* complemented strain, with survival monitored regularly. A noticeable difference in survival rates was observed between rats infected with the Δ*recA* strain and those infected with the wild-type strain, with higher survival noted in the former during the initial 5 days post-infection (Fig. 2A). Additionally, the impact of *recA* on *C. sakazakii* invasiveness was assessed by quantifying bacterial loads in blood, liver, and spleen through homogenization, plating, and enumeration. The Δ*recA* strain displayed significantly lower bacterial loads in these tissues compared to the wild-type strain, as depicted in (Fig. 2B through D). The phenotypic deficiency of the Δ*recA* strain was partially restored by introducing the *recA* plasmid.

### RecA knockout reduces desiccation tolerance and biofilm formation ability

**Fig 3:**
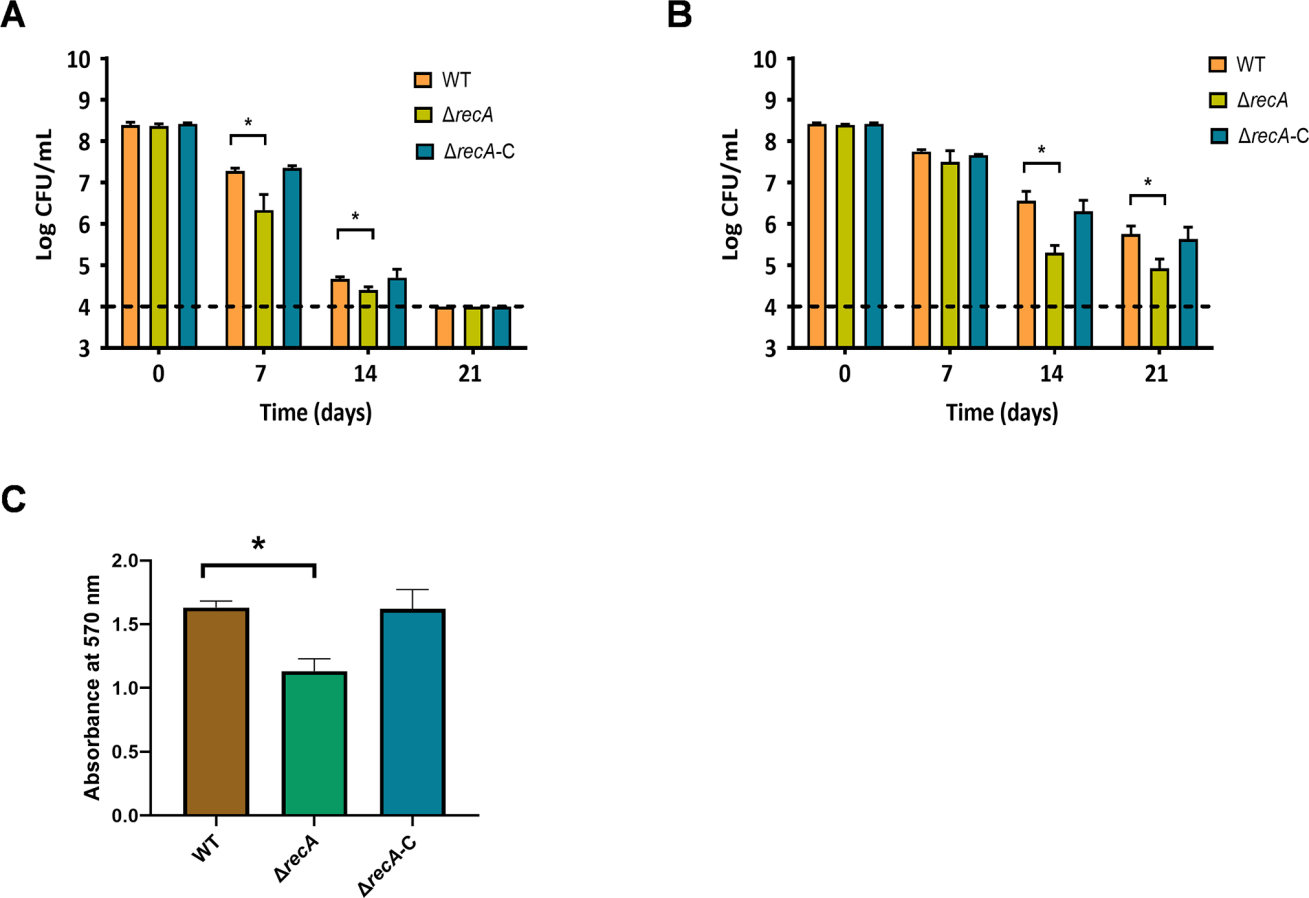
The impact of *recA* on the desiccation resistance and biofilm formation of *C. sakazakii* was assessed. (**A**) Quantification of surviving bacteria following 0, 7, 14, and 21 days of exposure to a dry environment through serial dilution and plating. (**B**) Detailed the survival of bacteria in dry infant formula powder over the same time periods. (**C**) Investigated bacterial biofilm formation over a 48-h period. The biomass adhered to glass substrates was assessed using crystal violet staining at 570 nm.

Desiccation tolerance was vital for *C. sakazakii* survival in infant formula. Tests were conducted to assess the role of *recA* in bacterial desiccation tolerance, revealing a significant decrease in survival rates of the *recA* knockout strain when exposed to dry air (Fig. 3A) or infant formula (Fig. 3B). Additionally, biofilm formation was crucial for bacterial environmental resilience. Tests were conducted to evaluate the role of *recA* in bacterial biofilm formation, showing a significant decrease in biofilm formation ability of the *recA* knockout strain compared to the wild-type and complemented strains (Fig. 3C). These findings highlight the importance of *recA* in both desiccation tolerance and biofilm formation in *C. sakazakii*.

### RecA knockout downregulates carbon metabolism proteins and impairs respiratory activity

**Fig 4:**
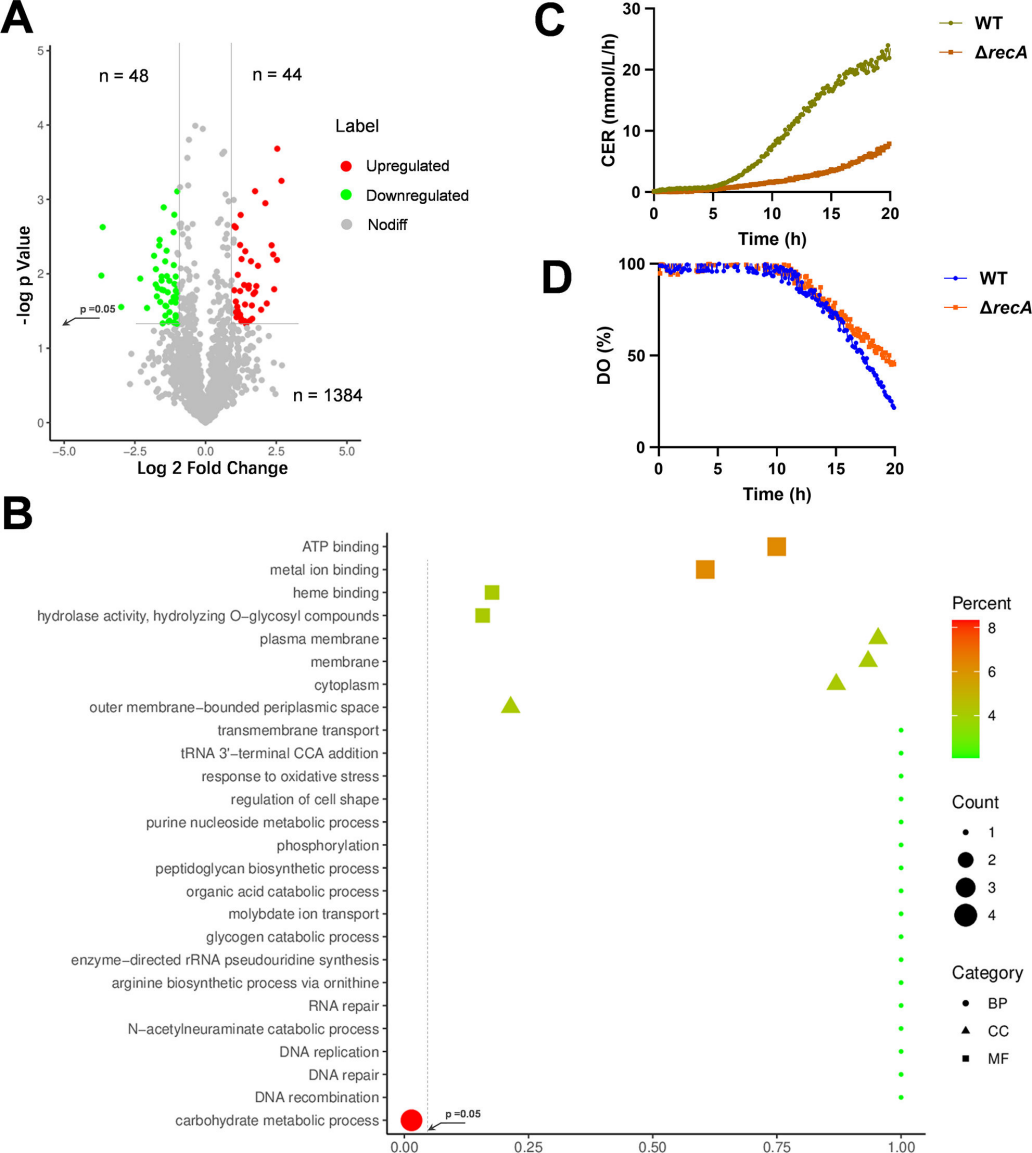
Impact of *reca* gene knockout on protein profiling. (**A**) Volcano plot depicting changes in protein abundance following *recA* gene knockout. Significant changes were operationally defined as a fold change exceeding 2 with a *P*-value below 0.05. Specifically, red dots denote proteins that have been significantly upregulated; green dots denote proteins that have been significantly down-regulated; and gray dots denote proteins that have not exhibited statistically significant differences. The quantity of proteins falling into each category was indicated within the graphical representation. (**B**) Gene Ontology analysis of proteins significantly upregulated due to the *recA* knockout categorized by biological process (BP), cellular component (CC), and molecular function (MF). Each shape’s size indicates the number of proteins in the category, while the color represents the percentage of proteins within that category. The x-axis indicates P-values, with higher values toward the right. The significance threshold (*P* = 0.05) was indicated on the plot. (**C**) Bacterial carbon dioxide evolution rate (CER) and dissolved oxygen (DO) consumption tests were conducted in triplicate, showing a representative set of data.

Proteomic analysis demonstrated that deletion of the *recA* gene resulted in significant upregulation of 44 proteins and downregulation of 48 proteins (Fig. 4A). While the upregulated and downregulated proteins did not exhibit enrichment in KEGG pathways, the downregulated proteins showed notable enrichment in biological processes associated with carbohydrate metabolic processes (Fig. 4B), indicating a potential adverse impact of *recA* deletion on carbohydrate metabolism. Further analysis of the impact of *recA* deletion on cellular energy metabolism revealed a reduction in carbon dioxide release (Fig. 4C) and dissolved oxygen (Fig. 4D) consumption and in the *recA* knockout strain, suggesting a notable decline in respiratory activity.

### Deleting RecA causes increased protein deamidation and oxidation

**Fig 5:**
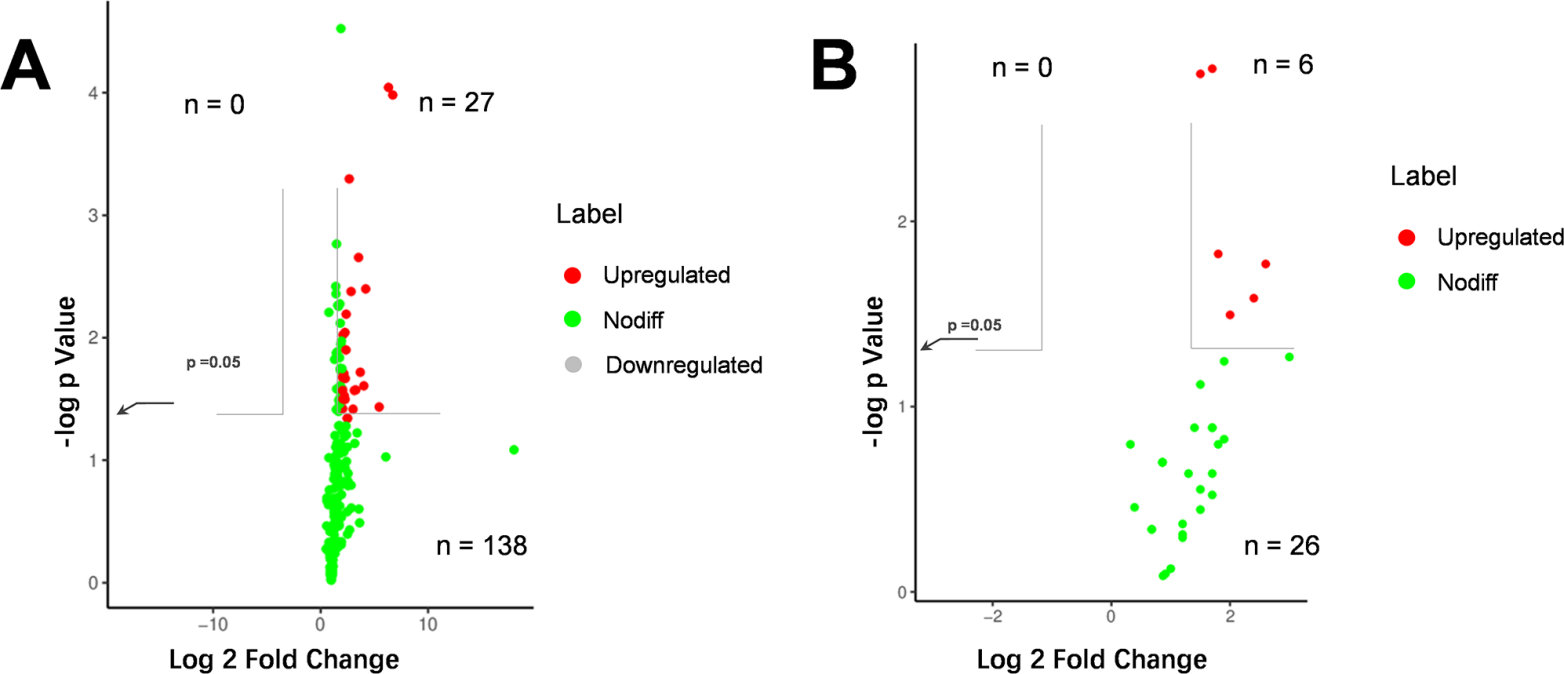
Effects of a *recA* gene knockout on protein modifications, specifically deamidation (**A**) and oxidation (**B**). Significant changes were determined by a fold change exceeding two and a P-value below 0.05. in the figure, red dots indicate upregulated proteins; gray dots indicate downregulated proteins; and green dots indicate proteins with no significant differences.

Previous research has identified a correlation between protein deamidation and the growth inhibition of *C. sakazakii* (13). Utilizing proteomic analysis, this study examined the potential protein deamidation resulting from the deletion of the *recA* gene (Fig. 5A). The analysis revealed that 196 proteins displayed deamidation modifications, with 27 proteins (13.8%) exhibiting a notable increase in deamidation following *recA* knockout. In contrast, no proteins demonstrated a significant decrease in deamidation post-*recA* knockout. Analysis of the 27 proteins exhibiting significantly increased deamidation as a result of *recA* knockout did not demonstrate significant enrichment in any KEGG or GO analysis. Furthermore, examination of the effects of *recA* gene deletion on protein oxidation through redox proteomics identified 32 proteins with oxidation modifications (Fig. 5B). Of the proteins analyzed, 26 demonstrated no statistically significant alteration in oxidation levels, whereas the remaining six proteins displayed a notable rise in protein oxidation modifications subsequent to the deletion of *recA*.

### Deleting RecA leads to functional impairment of fructose-bisphosphate aldolase

**Fig 6:**
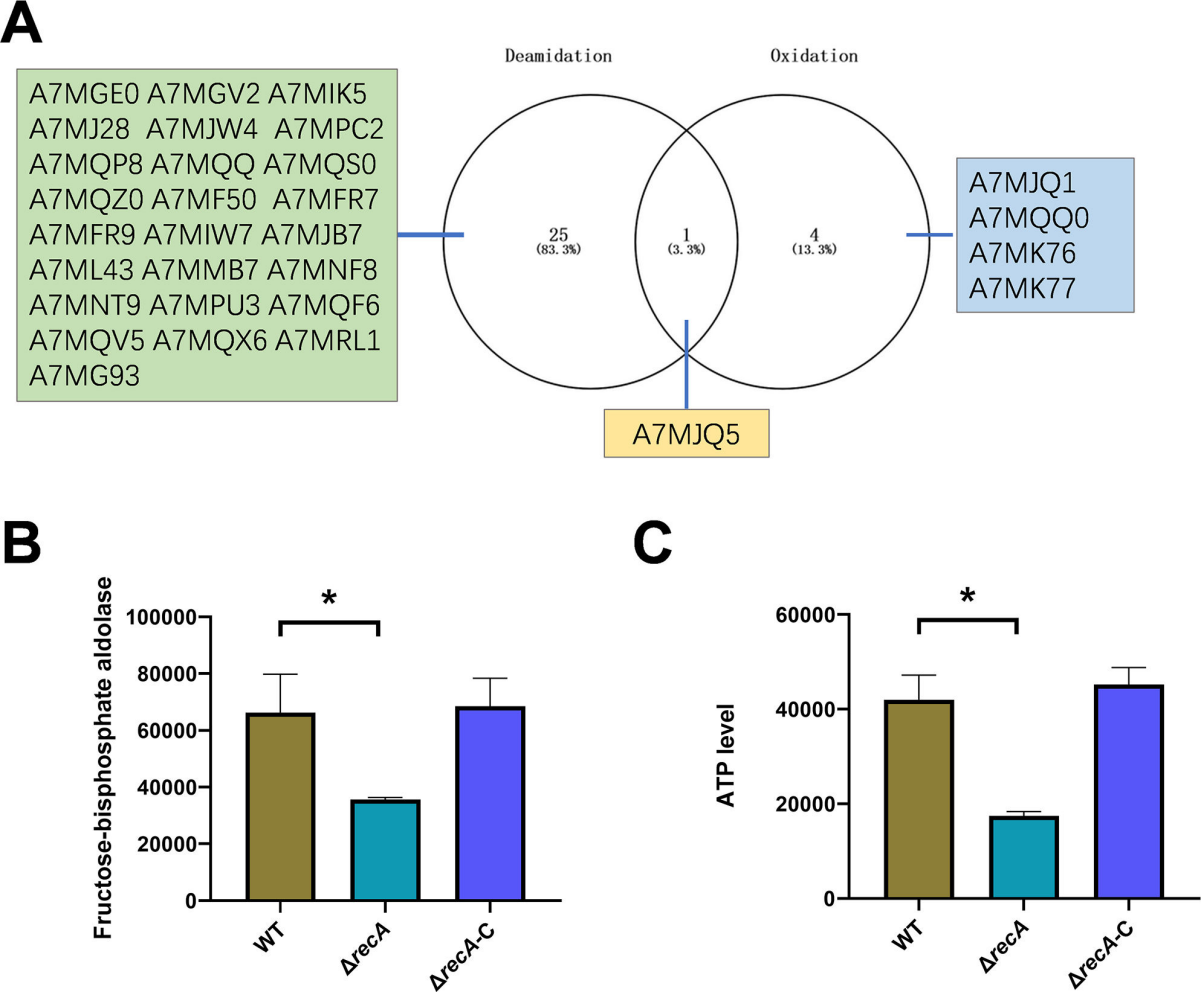
Impact of *recA* deletion on intracellular fructose-bisphosphate aldolase and ATP levels. (**A**) Venn diagram illustrating proteins with increased deamidation (yellow) and oxidation modifications in the Δ*recA* strain. The number of proteins in each category was indicated, with the intersecting proteins (identified by their UniProt accession numbers) listed. (**B**) Intracellular levels of fructose-bisphosphate aldolase in wild-type (WT), Δ*recA*, and complemented Δ*recA* strains. (**C**) Intracellular ATP concentration in wild-type (WT), Δ*recA*, and complemented Δ*recA* strains.

Analysis of Venn diagrams depicting proteins with deamidation and oxidation modifications identified fructose-bisphosphate aldolase (A7MJQ5) as exhibiting upregulation of both deamidation (specifically at the 63rd amino acid residue, asparagine) and oxidation (specifically at the first amino acid residue, methionine) in the *recA* knockout strain. Despite these modifications, the expression levels of fructose-bisphosphate aldolase remained unaltered according to the mass spectrometry analysis (data not shown). However, a reduction in the overall enzyme activity of intracellular fructose-bisphosphate aldolase was observed in the *recA* knockout strain, as depicted in Fig. 6B, along with a corresponding decrease in the ATP level, as demonstrated in Fig. 6C. These findings suggest that the deamidation and oxidation of fructose-bisphosphate aldolase may impede the protein’s typical functionality.

### Fructose-bisphosphate aldolase M1Q & N63D proxies for the oxidation of Met1 and the deamidation of Asn63 hamper enzyme activity

**Fig 7:**
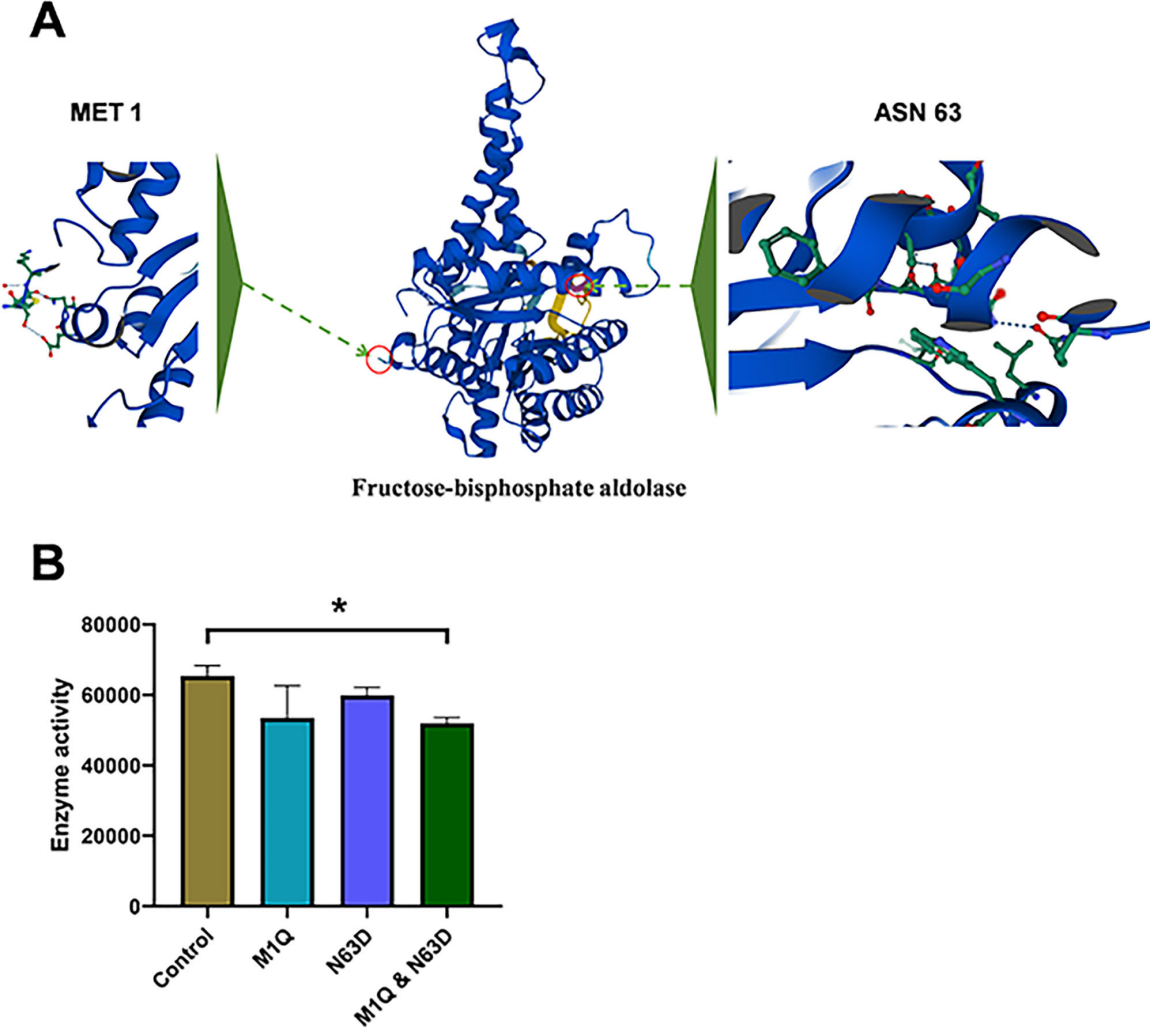
Impact of modifications on fructose-bisphosphate aldolase activity. (**A**) Structure of fructose-bisphosphate aldolase with modification sites. The left side highlights MET1 identified by mass spectrometry as an oxidation site, and the right side shows ASN63 identified as a deamidation site. Enlarged views of these modification sites were provided. (**B**) Effects of point mutations at the chemical modification sites, including M1Q, N63D, and M1Q & N63D, on the enzyme activity of fructose-bisphosphate aldolase. Proteins with point mutations were synthesized using a cell-free system.

**Fig 8:**
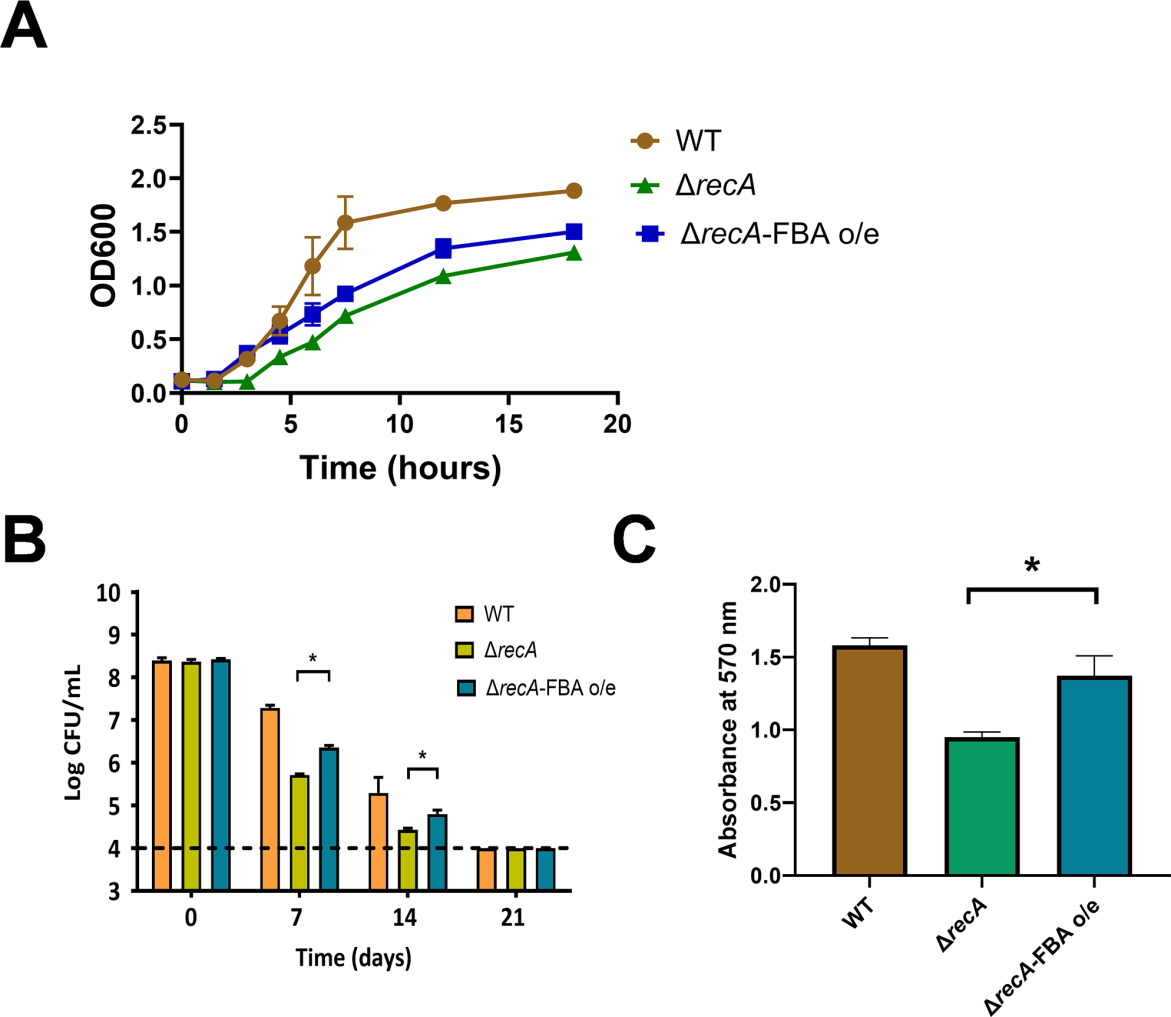
Impact of fructose-1,6-bisphosphate aldolase overexpression on bacterial adaptability. (**A**) Influence of FBA overexpression (FBA-o/e) on bacterial growth. (**B**) Quantifies bacterial survival after exposure to a dry environment for 7, 14, and 21 days, employing serial dilution and plating. (**C**) Investigates bacterial biofilm formation in stagnant conditions within vessels over 72 h, with biomass on glass substrates assessed via crystal violet staining at 570 nm.

Attempts to disrupt the coding gene of fructose-bisphosphate aldolase were met with repeated failure, indicating its likely indispensable role in *C. sakazakii*. Fructose-bisphosphate aldolase was then expressed *in vitro* using a cell-free system. An analysis of the enzyme activities of fructose-bisphosphate aldolase variants M1Q, N63D, and M1Q & N63D indicated that individual modifications at Met1 and Asn63 do not have a significant impact on enzyme activity (Fig. 7A and B). Nevertheless, the concomitant oxidation of Met1 and the deamidation of Asn63 lead to a notable decrease in the fructose-bisphosphate aldolase activity, suggesting that the oxidation of Met1 and the deamidation of Asn63 induced by *recA* knockout hinder the enzyme’s functionality (Fig. 7B). Subsequent experiments involving the overexpression of fructose-bisphosphate aldolase revealed a partial recovery of growth (Fig. 8A), desiccation tolerance (Fig. 8B), and biofilm formation abilities (Fig. 8C) in the *recA* knockout strain. These findings suggest that functional deficiencies resulting from oxidation and deamidation of the fructose-bisphosphate aldolase protein play a substantial role in the diminished adaptability of the *recA* knockout strain.

## DISCUSSION

RecA was a highly conserved protein across various organisms, including humans, bacteria, and plants, due to its essential functions in DNA repair and homologous recombination (29, 30). In pathogenic bacteria, RecA was crucial for bacterial survival and recognized as a significant target for combating bacterial infections (31, 32). Our investigation demonstrated that inactivation of RecA has a notable impact on the invasion and virulence of *C. sakazakii*. Similarly, studies on *Listeria monocytogenes* have shown that RecA activation was involved in the adhesion and invasion of Caco-2 intestinal epithelial cells (33). Moreover, the deficiency in adhesion to and invasion of Caco-2 cells observed in an in-frame RecA deletion mutant, along with the reduction in virulence of *L. monocytogenes* upon cleavage of RecA in bacteria, suggest a pivotal role for RecA in the pathogenic mechanisms of bacteria (34).

Our research has demonstrated that RecA has a significant impact on both desiccation tolerance and biofilm formation. Biofilms play a crucial role in enhancing bacterial resilience in adverse conditions, thereby promoting their survival (35). This discovery holds particular relevance in the context of food safety, as biofilms were essential for the environmental resilience of foodborne pathogens in processing facilities (36). Given the prevalence of *C. sakazakii* contamination in powdered milk and its potential for infection, understanding the mechanisms of biofilm formation and desiccation tolerance was of paramount importance in mitigating the spread of pathogenic bacteria (37). For instance, *A. baumannii* isolates that form biofilms have the ability to persist for prolonged durations in dry environments, thereby enhancing their resistance to antimicrobial agents and desiccation stress, which poses significant challenges for hospital patient care management (38). Research conducted on *Staphylococcus capitis* strains obtained from neonatal intensive care units demonstrated an increased capacity for adhesion and the formation of a sparse biofilm containing a higher concentration of viable and cultivable bacteria in comparison to strains originating from the alpha clone (39). Likewise, investigations on *L. monocytogenes* on surfaces used in mushroom processing indicated that the formation of biofilms and the ability to survive desiccation significantly influenced the efficacy of cleaning and disinfection procedures (40). Consistent with our research results, certain genes have been implicated in the regulation of both desiccation tolerance and biofilm formation. A recent study revealed that the absence of the *gsiD* gene in *C. sakazakii* disrupted the glutathione transport system, GsiABCD, leading to impaired glutathione import (41). This deficiency was correlated with diminished biofilm formation and decreased desiccation tolerance. Moreover, the gene ESA_RS15745 responsible for encoding a sugar transporter protein played a crucial role in regulating osmotic pressure in bacterial growth and reproduction and was also associated with desiccation tolerance and biofilm formation (42). Hence, genes that have a dual impact on desiccation tolerance and biofilm formation may present promising targets for the environmental management of *C. sakazakii*.

Proteomics research offers valuable insights into the impact of RecA on the phenotype of *C. sakazakii* (43). Our investigation revealed that the deletion of RecA results in the downregulation of proteins associated with carbon metabolism. Bacterial metabolism encompasses a range of processes, such as carbohydrate, amino acid, lipid, and nucleic acid metabolisms. Studies have shown that compounds like bacteriocin BM1157 and *p*-Coumaric acid can interfere with the normal metabolic functions of *C. sakazakii*, ultimately reducing bacterial viability and potentially causing cell death (44, 45). This suggested that metabolic pathways serve as promising targets for therapeutic intervention against this bacterium. Our investigation revealed that RecA serves as a pivotal regulator of carbohydrate metabolism, indicating that pharmaceutical agents aimed at RecA may exhibit enhanced antibacterial efficacy in conjunction with bacteriocin BM1157 and *p*-Coumaric acid.

Recent research has demonstrated a growing understanding of the relationship between protein post-translational modifications and the phenotype of *C. sakazakii* (46). Deamidation, a modification known to reduce protein activity, has been linked to various cellular processes (13). Our previous study revealed that *p*-Coumaric acid hindered the growth of *C. sakazakii* by promoting deamidation of bacterial elongation factors (43). Furthermore, deletion of *dnaK* resulted in increased deamidation of multiple proteins in *C. sakazakii*. In this study, we have identified a correlation between the *recA* gene and the process of protein deamidation and oxidation. Deletion of the *recA* gene resulted in an increase in protein deamidation and oxidation, particularly affecting the fructose-bisphosphate aldolase enzyme. Fructose-bisphosphate aldolase was a crucial enzyme in glycolysis, catalyzing the conversion of fructose-1,6-bisphosphate to glyceraldehyde-3-phosphate and dihydroxyacetone phosphate (47). Aldolase was ubiquitously present in all tissues and cells and plays a vital role in ATP production, particularly in glycolysis during anaerobic conditions (48). Research on fructose-bisphosphate aldolase has primarily concentrated on cancer cells due to its prevalent overexpression in a variety of cancers, such as squamous cell lung cancer, hepatocellular carcinoma, colonic cancer, osteosarcoma, and pancreatic cancer (49, 50). This heightened expression may be attributed to the heightened energy requirements essential for cancer cell invasion. Nevertheless, our study has uncovered that the deamidation and oxidation of particular sites within fructose-bisphosphate aldolase, triggered by the deletion of *recA*, can impair its enzymatic activity. This finding suggested that the growth, biofilm formation, and pathogenicity deficiencies observed in *recA* mutants may be attributed to decreased enzyme function of fructose-bisphosphate aldolase, leading to insufficient energy production.

## MATERIALS AND METHODS

### Bacterial strains, plasmids, and growth conditions

The study used specific bacterial strains, plasmids, and primers detailed in Tables 1 and 2. All bacterial strains were stored at −80°C and revived in LB medium before experimentation. Gene manipulation in *C. sakazakii* was done following established protocols using the pCVD442 suicide plasmid (51). Plasmid construction was achieved using a one-step seamless cloning method with a commercial kit (Vazyme, China) instead of traditional DNA digestion and ligation. Linearization of the pCVD442 plasmid was carried out by PCR using the primers pCVD442 fwd and pCVD442 rev. The gene fragments targeted for deletion were amplified using specific primers under the following conditions: denaturation at 95°C for 30 s, annealing at 60°C for 30 s, and extension at 72°C for 5 min, repeated for 34 cycles. The circularized plasmids were then transformed into *E. coli* S17 lambda pir chemically competent cells (Weidi, China) and selected on LB agar plates with 100 µg/mL ampicillin (Sangon, China). Deletion of genes in *C. sakazakii* was accomplished through *in situ* conjugation with *E. coli* S17 lambda pir, followed by antibiotic selection and counter-selection with sucrose. The deletions were verified through PCR and sequencing with external primers. The pACYC184 plasmid was utilized for complementing the gene deletion strains (52). Overexpression of the fructose-bisphosphate aldolase strain was achieved by replacing the original promoter of the complementation gene on the pACYC184 plasmid in the complementation strain with the lac promoter, followed by high-level expression induced by IPTG.

**TABLE 1:** Bacterial strains and plasmids used in this study

Strains, plasmids	Description	Reference, source
*C. sakazakii*		
WT	Wild-type *C. sakazakii* BAA-894	(13)
Δ*recA*	Markerless deletion mutant Δ*recA*	(43)
*E. coli*		
S17 λpir	Strain for construction harboring lambda pir	(12)
S17 lambda pir-Δ*recA*	S17 λ pir harboring pCVD442-Δ*recA*	(43)
DH5α	Strain for construction	(13)
DH5α-*recA*	DH5α harboring pKC1139-*recA*	(43)
Plasmids		
pACYC184	Low-copy plasmid	(12)
pACYC184-*recA*	*recA* complementation vector	(43)
pCVD442	Suicide plasmid for markerless deletion	(43)
pCVD442-Δ*recA*	*recA* deletion plasmid	(43)

**TABLE 2:** Primers used in this study

Primers	Sequence (5′−3′)
For construction	
pCVD442-fwd	GGCTGTCAGACCAAGTTTACTCATATATACTTTAGATTG
pCVD442-rev	GCAGATACTCTTCCTTTTTCAATATTATTGAAGCATTTATCAG
Δ*recA*-A	GAAAAAGGAAGAGTATCTGCGGATAACCATAGTACGCACTATG
Δ*recA*-B	CTGCATCAGCAGCCCTTGAGATTACATTTTTACTCCTGTCATGC
Δ*recA*-C	TAATCTCAAGGGCTGCTGATGCAG
Δ*recA*-D	GATTAATTGTCAAGGCTAGCGATGATTATTCGGGACCAGAGAGCTA
For sequencing confirmation	
Δ*recA*-E	CCCGCCAACGGCCAGGCGCCG
Δ*recA*-F	TGAAGTTGCCCAAGCATTTCGAAGAAGGT

### Growth curve determination and viable cell count

The growth curves of the wild-type (WT), Δ*recA* mutant, and complemented ΔrecA strains (Δ*recA*-C) of *C. sakazakii* were assessed in LB medium at pH 7.0. Bacterial cultures were initially grown overnight at 37°C with shaking (200 rpm), then diluted 1:100 in fresh LB medium to a starting optical density at 600 nm (OD600) of approximately 0.1. Bacterial growth was monitored by measuring OD600 at 1-h intervals over an 18-h period using a spectrophotometer (Eppendorf). To determine the viable cell count, samples were collected at specific time points and serially diluted in phosphate-buffered saline (PBS), and 50 µL of each dilution was spread onto LB agar plates. Plates were incubated at 37°C for 24 h, after which colony-forming units (CFUs) were counted and expressed as CFUs per mL.

### Rat survival assay

The rats were administered oral gavage according to previously established protocols (43). An inoculum of 10^9^ bacteria was delivered, and survival was monitored daily thereafter. At 24 h post-infection, the bacterial load in the rats’ organs was assessed via organ homogenization, serial dilution plating, and colony counting on agar plates. Each organ was rinsed with saline, weighed to a standardized weight, and placed in a thick-walled 2 mL centrifuge tube with 0.5 mL of cold PBS and steel beads. The organs were homogenized by oscillating at 6000 rpm for two 15-s intervals, resulting in a fully homogenized liquid sample. Each sample was then diluted five times in PBS and plated on LB agar plates. CFUs were counted after overnight incubation at 37°C.

### Desiccation resistance assay

The *C. sakazakii* BAA-894 strains preserved at −80°C were introduced into LB medium (Oxoid, UK) and incubated in a shaking incubator at 37°C for 16–18 h. Following this, the culture was diluted 1/100 and introduced into 10 mL of LB medium until achieving an optical density of 0.6–0.8 at 600 nm to generate a bacterial suspension (43). The bacteria were subsequently centrifuged, resuspended in PBS or reconstituted infant formula powder with water, and transferred to a sterile 96-well plate for desiccation at 37°C. To determine the initial cell count, 100 mL of each bacterial suspension was serially diluted in PBS and plated on agar for culturing. To assess the viability of bacteria following desiccation, on Days 7, 14, and 21 of drying, saline was added to the wells of the 96-well plate. The cells were dislodged by repeated pipetting and scraping, followed by serial dilution and plating onto Luria–Bertani (LB) agar for enumeration. Following an incubation period of 24 h at 37℃, the colony-forming units present on the agar plates were quantified. Each experimental procedure was replicated thrice for validation.

### Biofilm formation assay

The biofilm formation ability of *C. sakazakii* BAA-894 strains was assessed using 96-well polystyrene microtiter plates, as described previously (53). Bacterial cultures were incubated under static conditions at 28°C for 48 h in tryptic soy broth (TSB) supplemented with 0.25% glucose, ensuring conditions optimal for biofilm development. Incubations were performed in a humidity-controlled incubator, with a water basin placed inside to maintain a consistent moisture level. Following incubation, non-adherent cells were removed by gently washing the wells three times with sterile deionized water. The adherent biofilm biomass was stained with 1.0% crystal violet solution for 30 min at room temperature. Excess dye was rinsed off by washing three times with sterile deionized water. The bound crystal violet was solubilized with 33% (v/v) acetic acid for 30 min, and the absorbance was measured at 570 nm using a microplate reader (ShanPu, China). Sterile TSB served as the negative control, and the optical density (OD) values of the biofilm were calculated by subtracting the OD of the negative control from the OD of the test wells. Each strain was tested in triplicate, and the results were expressed as the mean OD570 nm ± standard deviation.

### Respiratory activity

Respiratory activity was assessed using established methods from previous study (32). Dissolved oxygen (DO) levels were measured with DO sensors (Hamilton, Switzerland). Real-time monitoring of CO_2_ concentrations was conducted using mass spectrometry coupled to the off-gas line (Thermo Scientific, USA). Furthermore, off-gas CO_2_ data were analyzed to determine the carbon dioxide evolution rate (CER).

### Protein synthesis

*E. coli* lysate was utilized as the enzyme system for cell-free protein synthesis (Promega, USA), in which the full-length gene, including the promoter, was initially amplified through PCR. Point mutations were introduced by incorporating mutation sites into the amplification primers. In cases where multiple fragments were required, fusion PCR was employed to merge them into a complete segment. The resulting PCR products were purified and confirmed by Sanger sequencing using the primers as sequencing primers and subsequently added to the cell-free protein synthesis system as per the manufacturer’s guidelines. Following the completion of the reaction, proteins were purified using ultrafiltration tubes and quantified using a BCA assay (Thermo Scientific, USA).

### Protein extraction and digestion

The bacterial protein extraction for proteomic analysis followed previously described methods (13, 43, 54, 55). *C. sakazakii* cells were cultured in LB medium overnight, pelleted, washed with PBS buffer, and resuspended in B-per protein extraction reagent (Thermo, USA) with a protease inhibitor cocktail (Beyond, China). The supernatant was filtered through a 0.22 µm filter to get a protein solution, and the protein concentration was measured using a BCA analysis kit. A mixture of 100 µg protein, 1 µL of tris(2-carboxyethyl) phosphine (Thermo Scientific, USA), 1 µL of iodoacetamide (Thermo Scientific, USA), and guanidine hydrochloride (Thermo Scientific, USA) was prepared in a total volume of 100 µL. After mixing, the mixture was incubated in the dark at room temperature for 40 min. The sample underwent centrifugation and buffer replacement with ammonium bicarbonate solution in a 10 kD ultrafiltration tube. Then, 2 µg of recombinant trypsin (Thermo Scientific, USA) was added and the sample was incubated overnight at 37°C to fully digest the proteins into peptides. The peptide samples underwent centrifugation for collection, followed by drying in a vacuum centrifuge, dissolution in a 0.1% formic acid solution, and subsequent analysis via mass spectrometry.

### LC–MS/MS analysis

Mass spectrometry data were acquired using established methods (54), with protein samples analyzed using a Q Exactive Plus mass spectrometer (Thermo Scientific, USA) after trypsin digestion. Chromatographic separation was done on a C18 column with a flow rate of 0.5 mL/min and a mobile phase of 0.1% formic acid solution and acetonitrile. Mass spectra were analyzed using Maxquant software and matched against the UniProt database for *C. sakazakii* BAA-894. Analysis parameters included fixed modification of carbamidomethylation of cysteine and variable modifications of deamidation and oxidation, with allowance for up to two missed cleavage sites per protein. Protein and peptide identifications were based on a maximum false discovery rate (FDR) of 1.0%, and protein quantification was normalized by median values.

### Enzymatic activity of fructose-bisphosphate aldolase and the quantification of ATP

The activity of fructose-bisphosphate aldolase was measured using a spectrophotometric assay kit (Solarbio, China) (56) following the manufacturer’s instructions. In this assay, fructose-bisphosphate aldolase catalyzed the conversion of fructose-1,6-bisphosphate into glyceraldehyde-3-phosphate and dihydroxyacetone phosphate. The reaction was coupled with the activities of triosephosphate isomerase and α-glycerophosphate dehydrogenase, which converted NADH and dihydroxyacetone phosphate into NAD and α-glycerophosphate, respectively. The decrease in absorbance at 340 nm, corresponding to the oxidation of NADH to NAD, was monitored to reflect the activity level of fructose-bisphosphate aldolase. All reactions were performed at 37°C in a buffer containing 50 mM Tris–HCl (pH 7.4), 5 mM MgCl2, and 1 mM fructose-1,6-bisphosphate. ATP concentrations were determined using an ATP Assay Kit (Abcam, USA). The assay quantified ATP by measuring the phosphorylation of glycerol, which was detected fluorometrically at excitation/emission wavelengths of 535/587 nm. The reactions were carried out in 96-well plates, and fluorescence was measured using a microplate reader (BioTek, USA).

### Statistical analysis

The experiments were independently performed three times to validate the findings. Bioinformatics analysis, including Gene Ontology (GO) and Kyoto Encyclopedia of Genes and Genomes (KEGG) pathway analysis, utilized data from the DAVID online database (57; https://david.ncifcrf.gov). Statistical analysis was conducted using GraphPad Prism software (version 8.3). Differences were assessed using the Student’s *t*-test, with statistical significance set at *P* < 0.05.

## Data Availability

All datasetsdata sets produced in this study were provided within the manuscript and/or supplementary files. Additionally, the MS proteomics data have been deposited in iProX and can be accessed using the accession number IPX0005984000 .
